# Expression of the novel serum biomarker of testicular germ cell tumours miR-371a-3p in serum of pregnant women: a case–control study

**DOI:** 10.1186/s40001-025-02906-8

**Published:** 2025-08-02

**Authors:** K. P. Dieckmann, M. Hubert, V. Ragosch, C. Kern, B. Hansen, M. Klemke, G. Belge

**Affiliations:** 1 Department of Urology, Asklepios Klinik Altona, Hamburg, Germany; 2 Faculty of Biology and Chemistry, University of Bremen, Bremen, Germany; 3 Maternity Department, Asklepios Klinik Altona, Hamburg, Germany; 4 mir|detect GmbH, Bremerhaven, Germany; 5 Faculty of Biology and Chemistry, Department of Tumour Genetics, University of Bremen, Leobener Strasse 2, FVG-Ost, 28359 Bremen, Germany

**Keywords:** Testicular germ cell tumour, Pregnancy, MicroRNA-371, Tumour marker, Embryogenesis, Stem cells

## Abstract

**Background:**

The microRNA-371a-3p (M371) is a sensitive novel serum biomarker of testicular germ cell tumours (GCTs) and a certified test is available for consistent clinical testing. In view of the well-known biological analogies of GCTs and embryogenesis, we hypothesized that the marker substance M371 is also present in serum of pregnant women. The goal of this report was to analyse maternal serum for M371.

**Materials and methods:**

M371 serum levels were measured in 36 third-trimester pregnant women. Control groups consisted of 12 non-pregnant young women, 12 healthy young males, and 12 patients with GCTs. M371 levels were measured by quantitative real time PCR using the certified M371 test with the standard cutoff of RQ = 5. Statistical methods involved receiver operating characteristics (ROC) analysis with Youden index analysis, and statistical comparisons of median serum levels of patients with those of controls as well as for comparisons of subgroups of patients according to age and infant sex.

**Results:**

All pregnant women had measurable M371 levels, with 83.3% of the patients having elevated levels above the cutoff, while traces below cutoff were detected in the remainder. Healthy female and male controls were both below cutoff. ROC analysis revealed a 100% sensitivity and 100% specificity of the test when the cutoff of RQ = 0.4 defined by Youden index analysis was employed. The median level in pregnant women was significantly lower than that in GCT patients (10.8 [interquartile range 6.1–20.3] versus RQ = 139.5 [IQR 54.9–630.3], *p* < 0.001). Individual M371 levels were not associated with patient age and with infant sex.

**Conclusions:**

The evidence for elevated levels of microRNA-371a-p in maternal serum is a novel finding. This result accords with the various analogies between GCTs and embryogenesis documented previously. The finding supports the view that cells involved in human reproduction share epigenetic features with human embryonic stem cells. Further studies are required to explore if this finding could be utilized clinically.

**Supplementary Information:**

The online version contains supplementary material available at 10.1186/s40001-025-02906-8.

## Introduction

Testicular germ cell tumours (GCTs) and human embryogenesis exhibit a number of similarities, morphologically and biochemically [1]. Typical GCT tissue patterns resemble embryonic features, morphologically and immunohistochemically, which is witnessed by the terminology of GCT subtypes, such as embryonal carcinoma, chorio carcinoma, and yolk sac tumour [2]. Biochemically, GCTs may produce substances, such as beta human chorionic gonadotropin (bHCG) and alpha fetoprotein (AFP) [3] that are also physiologically encountered during embryogenesis in pregnancy and that are employed for monitoring pregnancies, clinically. Noteworthy, the same two substances were found to serve as serum biomarkers of GCTs [4], and not surprisingly, a gynaecologist was the first to discover this particular role of bHCG in 1932 [5].

Currently, a novel serum biomarker of GCTs, the microRNA (miR)−371a-3p is at the verge of demonstrating its usefulness in clinical practice [6, 7]. The physiological role of this and allied miRs (miRs-372-373; 302; 367) is to control pluripotency in human embryonic stem cells [8–10]. Accordingly, this miR is down-regulated in adult human cells.

In view of the various biological analogies between embryogenesis and GCTs, it is rational to assume epigenetic c analogies to exist, too. We, therefore, analysed sera of pregnant women to prove the hypothesis that miR-371a-3p serum levels in this population are higher than in controls.

## Patients and methods

Serum samples were derived from consecutive pregnant women attending the maternity department of Asklepios Klinik Altona, Hamburg, for preparing delivery in 2024. Due to institutional regulations, only patients with third-trimester pregnancies were available for this pilot study. Only patients > 18 years with a normal course of pregnancy were included. Exclusion criteria involved age > 45 years, concomitant disease, and mental handicap. The following clinical data were registered: patients age, gestational age, infant sex (male/female). For comparison, three control groups were included: (1) age matched non-pregnant women, (2) healthy males, and (3) consecutive patients treated for testicular GCT clinical stage 1 in Asklepios Klinik Altona in 2024.

Serum levels of microRNA-371a-3p were measured as described earlier [11]. Briefly, the IVDR-certified M371-Test (mir|detect, Bremerhaven, Germany) was employed for quantitative real time polymerase chain reaction (qPCR) using miR-30b-5p as endogenous control. Results were provided as relative quantities (RQ values) according to the ∆∆Ct method [12]. Due to the lack of information on M371 expression in pregnant women, a cutoff value of RQ = 5 was assumed which is the standard for primary diagnosis of GCTs with the test [11].

All patients gave written informed consent. Ethical approval was provided by the Ethical Committee of Ärztekammer Bremen (HR/RE-301A). All study activities were conducted in accordance with the Declaration of Helsinki of the World Medical Association, amended by the 64th General Assembly in October 2013.

Statistical analysis involved calculating median levels with interquartile ranges (IQRs) of M371 levels as well as percentages of patients with miR levels above cutoff in the entire group of pregnant women, in subgroups thereof according to patient´s age, infant sex, and gestational age, and in control groups. The Mann–Whitney *U* test was employed for statistical comparisons across subgroups and controls. Statistical significance was assumed at *p* < 0.05. Receiver operating characteristics (ROC) analysis was performed with calculating the Youden index to determine the ideal cutoff value for distinguishing pregnant subjects from non-pregnant women. Statistical evaluation was performed using IBM SPSS Statistics for Windows, Version 29.0.2.0 (IBM Corp., Armonk, NY).

## Results

**Fig. 1:**
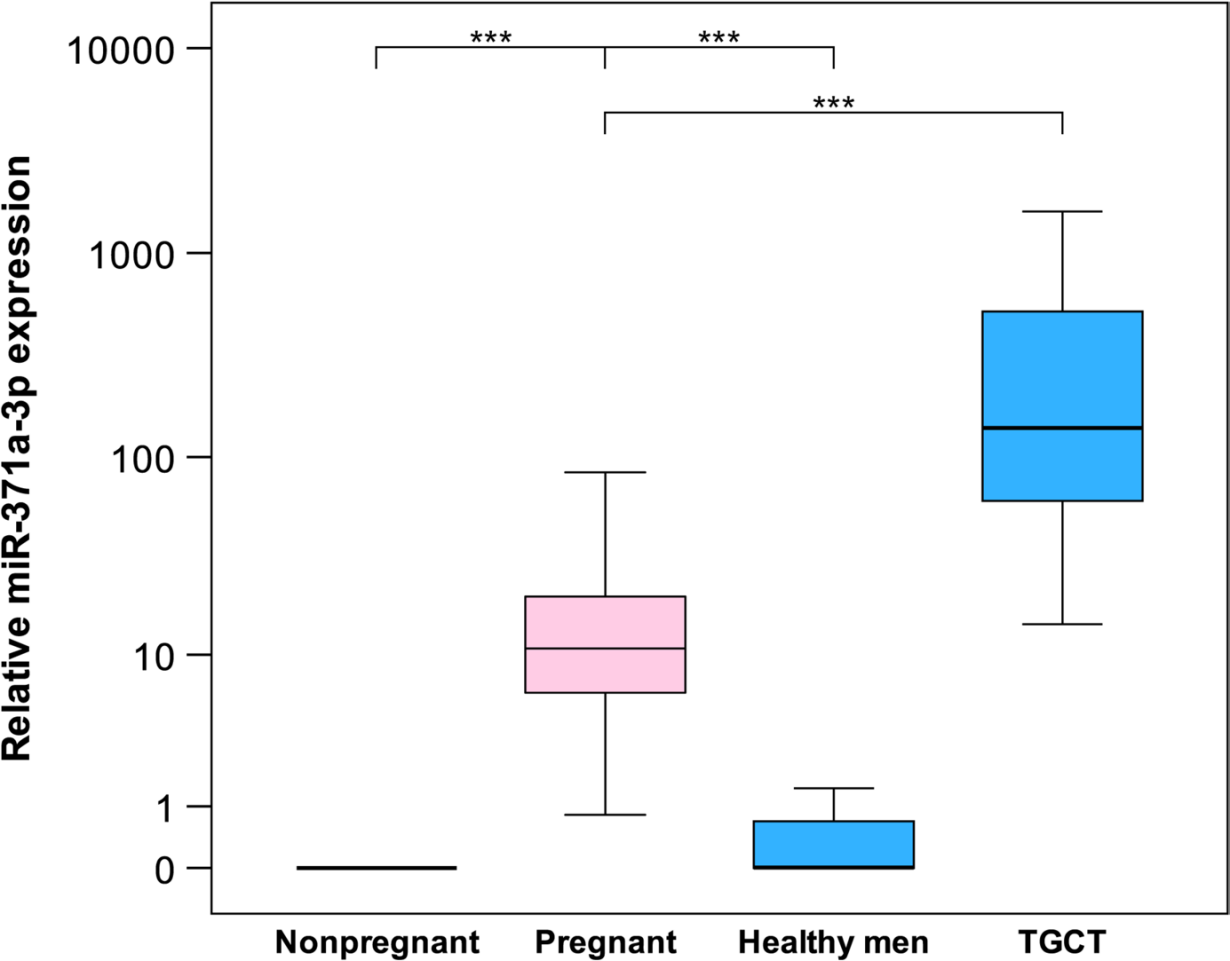
Relative miR-371a-3p serum levels in pregnant women and controls. Box plot illustration showing the** r**elative miR-371a-3p expression in serum of non-pregnant women (*n* = 12), pregnant women (*n* = 36), healthy men (*n* = 12) and men with testicular germ cell tumours (TGCT, *n* = 12). The miR-371a-3p expression in pregnant women is significantly higher than in non-pregnant women and healthy men (controls) but significantly lower than in men with TGCTs (***p < 0.001). The *y*-axis is depicted in a logarithmic scale

**Fig. 2:**
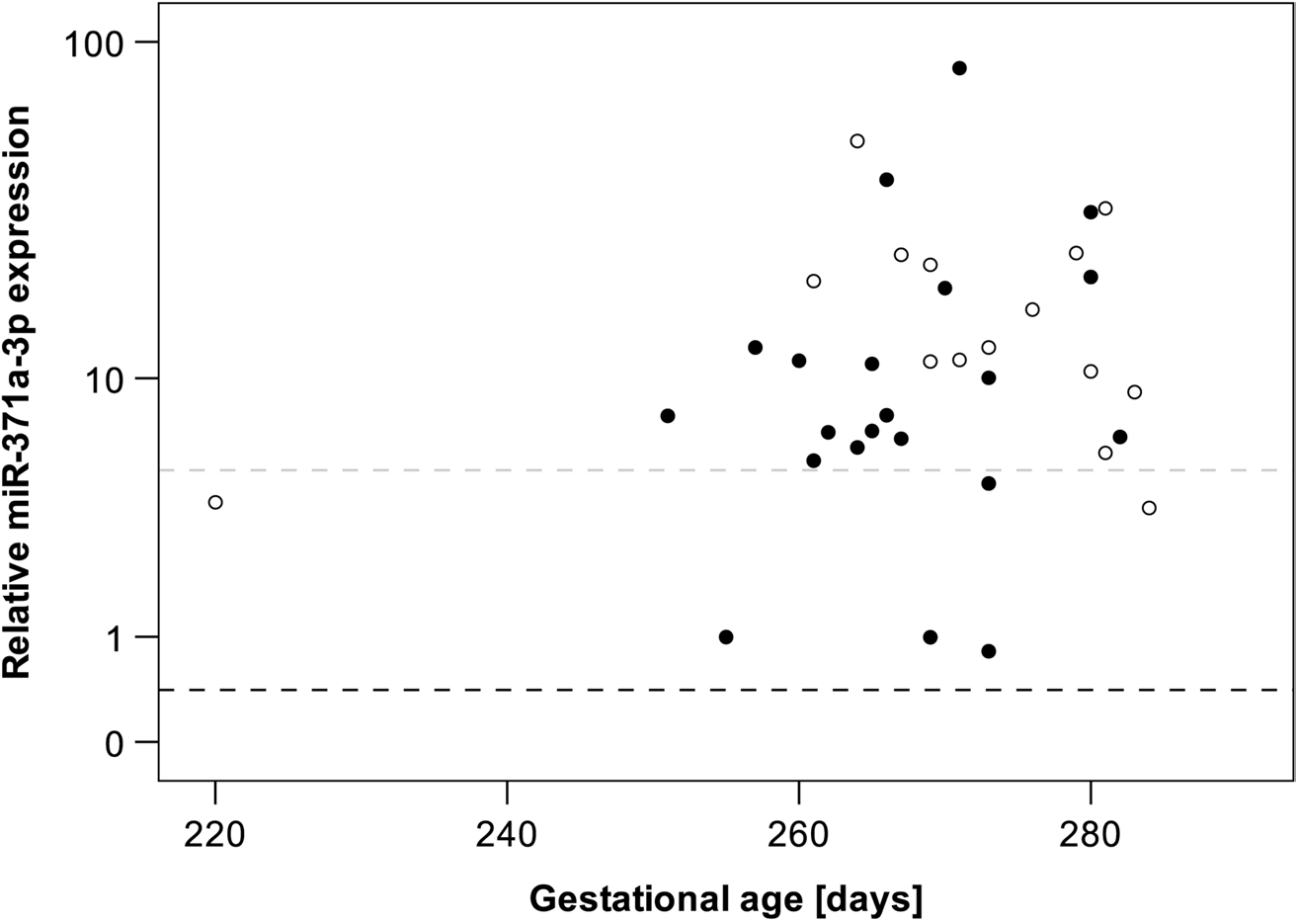
Individual serum levels of miR-371a-3p in pregnant women (*n* = 36) in correlation to gestational age. The grey dashed line indicates the standard cutoff value (RQ = 5), whereas the black dashed line indicates the cutoff defined by Youden index analysis of the current data (RQ = 0.409). Filled circles: male infant; open circles: female infant. The *y*-axis is depicted in a logarithmic scale

A total of 36 pregnant women were included. Median age was 33.5 years (range 26–42 years). All were in the third trimester of pregnancy with a median of 269 days (range 220–284). While miR-371a-3p was completely undetectable in all 12 non-pregnant women, it was detected in all 36 pregnant women. However, in six pregnant women the observed relative expression was below the standard cutoff value (RQ = 5). Thus, 83.3% of pregnant women had elevated miR371a-3p serum levels with a median M371 expression of RQ = 10.8 (IQR 6.1–20.3). Youden index analysis revealed a cutoff value of RQ = 0.4 to allow identifying pregnant women. Using this cutoff, all pregnant women were detectable corresponding to both 100% sensitivity and specificity of the test, and the area under the ROC curve would be the ideal value of AUC = 1.0. In healthy males (*n* = 12), the median RQ was below cutoff, while the M371 level was significantly higher in GCT patients (*n* = 12; RQ = 139.5; *p* = 0.001) (details in Table 1, Fig. 1 and Suppl. Tables S1, S2, S3). Patient age and infant sex was not associated with serum M371 levels. As all participants were in late stages of pregnancy, associations of RQ values with gestational age could not be analysed (Table 1 and Fig. 2).

**Table 1:** Clinical data and relative expression values of miR-371a-3p in serum of pregnant women

Case ID	Age [years]	Gestational age [days]	Gravidity	Parity	Fetal sex [male/female]	RQ
1	26	220	2	1	Female	3.855
2	32	280	1	1	Male	20.446
3	34	257	2	1	Male	12.468
4	31	283	2	1	Female	9.043
5	39	273	3	2	Female	12.468
6	41	264	3	2	Female	51.557
7	36	262	1	0	Male	6.703
8	37	276	2	0	Female	16.294
9	34	273	4	1	Male	0.818
10	32	261	1	0	Female	19.867
11	33	273	1	0	Male	4.496
12	33	266	2	1	Male	39.708
13	41	281	3	1	Female	5.724
14	34	279	1	0	Female	24.105
15	35	266	4	2	Male	7.617
16	38	282	1	0	Male	6.468
17	31	270	2	1	Male	18.926
18	42	255	5	2	Male	0.997
19	40	271	3	2	Female	11.407
20	34	269	2	1	Male	0.995
21	28	264	2	1	Male	5.971
22	37	260	2	1	Male	11.347
23	34	280	2	0	Male	31.882
24	33	265	3	1	Male	11.092
25	31	269	2	1	Female	11.28
26	41	267	1	0	Male	6.383
27	27	251	3	2	Male	7.581
28	31	273	2	1	Male	10.03
29	36	267	2	1	Female	23.816
30	28	269	2	1	Female	22.235
31	26	265	1	0	Male	6.769
32	33	281	1	0	Female	32.713
33	35	284	3	1	Female	3.678
34	27	280	1	0	Female	10.506
35	33	271	1	0	Male	83.981
36	29	261	2	1	Male	5.389

*RQ* relative quantity

## Discussion

This study provided clear evidence for elevated serum levels of miR371a-3p in the majority of third-trimester pregnant women.

This is a novel finding. A flurry of studies had investigated microRNAs in pregnancy to date [13, 14] and one main finding was that microRNAs of the chromosome 19 microRNA cluster (C19MC) were present in maternal plasma in various stages of pregnancy [15–17]. In addition, several potentially relevant associations of microRNA-dysregulations with pregnancy disorders had been revealed [18, 19]. The cluster miR-371-3 is also mapping to chromosome 19 close to the C19MC locus (19q13.42) and these miRNAs have been found to be expressed in placental tissue [20, 21].

Gu et al. reported miR-371-5 to be stronger expressed in placental tissue of first trimester pregnancies than in that of third trimester [22]. Although that miR belongs to the miR-371-3 cluster, it is different from microRNA-371a-3p, the one analysed in this study. In essence, the Gu study found a much higher expression of the miR in early pregnancies, but it did not report a non-expression in late pregnancy. As the M371 test used in this study involves a very high sensitivity, the results of the Gu study and of the present one are probably not conflicting particularly, because the elevation of serum levels found in the present study were mostly of modest extent.

Some studies suggested the microRNAs of the miR371-3 cluster to be present in the maternal circulation, too [16]. So far, only one study briefly reported on measuring of these circulating in four pregnant women, however, the results were not detailed [21].

The present study found expression of microRNA-371a-3p in serum of all pregnant women, whereas it was undetectable in non-pregnant women. In 83% of third-trimester pregnancies the relative expression of miR-371a-3p exceeded the standard cutoff value of RQ = 5, albeit with modest extent in the majority of cases.

However, if the ideal cutoff of RQ = 0.4 as revealed with Youden index analysis was employed, virtually all pregnant women were identified with the test. In addition, even with an empirically defined cutoff of RQ = 2, the test sensitivity would still be 92%.

The extent of M371 elevations in maternal serum is much lower than in GCT patients [11, 23]. In addition, microRNA-371a-3p levels in maternal serum are much lower than those of the miRs from C19MC, and therefore, previous studies using standard PCR methods did not detect this miR in the maternal circulation [15, 16]. However, during the investigation of microRNA-371a-3p as a serum biomarker for GCTs, a highly sensitive test was developed using a preamplification step allowing for detection of low amounts of the miR in serum [24]. Using this sensitive test, clear evidence for the presence of miR-371a-3p in maternal serum was unequivocally documented in the present study.

Noteworthy, the extents of M371 serum level elevations found in pregnant women exhibit a great variability with a range from 83.98 to 0.818 (ratio 102.7). The underlying biological reasons for the great differences of M371 elevations remain elusive. Associations with patients age, gestational age, fetal sex and number of preceding pregnancies were ruled out. However, putative associations of M371 levels with pathologic conditions of pregnancy as found with other microRNAs [25] could not be assessed, because the number of patients enrolled in this pilot study was too small. Strikingly, the variability of RQ values is likewise great in GCT patients with a range of 1594.62–14.52 (ratio 109.8), although the GCT population was quite homogeneous, since only patients with clinical stage I disease had been included. Thus, variability of M371 measurement results in pregnant women could also relate to non-pregnancy-related factors including technical issues.

The detection of this miR in maternal serum highlights another analogy between GCTs and physiological characteristics of human reproduction [26]. Epigenetic elements can now be added to the hitherto known similarities relating to morphological, immunohistochemical [1, 2], and biochemical features [27]. The specific site of origin of miR-371a-3p in pregnant women remains unknown. Likewise, the biological role of microRNA-371a-3p in maternal serum is still incompletely understood despite the numerous reports on the complex epigenetic signature of pregnancy [28–31]. While this miR has been documented in placental tissue, its presence in immature foetal tissues is unexplored. As miRs 371-3 are characteristic for embryonic stem cells, it might be hypothesized that a small number of such stem cells are still present during foetal development or that the expression of these miRs is not completely downregulated in developing tissues. Accordingly, one might expect higher levels in maternal serum in first trimester pregnancies when the developments of foetal organs are still at earlier stages. In addition, twin pregnancies might have higher serum levels of M371.

Biologically, the present finding also aligns with the previous detection of miR-371a-3p in the tissues of male reproductive organs [32, 33], and in seminal plasma [34, 35]. These studies demonstrated abundant presence of miR-371a-3p in tissues and body fluids, where germ cells or their direct outgrowths are present, i.e., testicular parenchyma and epididymal tissues but not in adjacent structures, such as intratesticular connective tissue, prostate or urethra. These studies provided evidence for the understanding that miRNA-371a-3p is not only present in embryonic stem cells, where its original place is supposed to be and in GCT cells mimicking stem cell features but also in cells and tissues involved in human reproduction and development. Based on these observations, the assumption follows that cells involved in reproduction and development probably share epigenetic features particularly the microRNA-profile with embryonic stem cells.

Limitations of the study relate to the overall small number of subjects analysed and to the lack of examinations in earlier periods of pregnancy or thereafter. However, the present study was merely scheduled as an exploratory pilot study that in fact confirmed the study hypothesis.

## Conclusion

Elevated levels of microRNA-371a-p in maternal serum have not been reported before. The expression of this stem-cell associated microRNA accords with the various biological analogies between GCTs and embryogenesis already known. The finding supports the view that cells involved in human reproduction share epigenetic features with human embryonic stem cells. Further investigations are required to corroborate the promising results. In particular, it appears worthwhile exploring the expression of this miR and allied miRs of the cluster during the full course of pregnancy and thereafter to better understand the biological background of the presence of microRNA-371a-3p in pregnancy. Furthermore, in view of the known associations of other microRNAs with pregnancy disorders [36, 37], it appears worthwhile to evaluate the serum levels of this miR in pathologic conditions of pregnancy. A vision could be that miR-371a-3p and possibly also allied miRNAs might offer a novel tool for monitoring pregnancy with early detection of gestational dysregulations in a non-invasive way.

## Supplementary Information

Supplementary material 1. Table 1. Clinical data and relative expression of miR-371a-p in serum of non-pregnant women. Table 2. Clinical data and relative expression values of miR-371a-3p in serum of patients with germ cell tumours clinical stage 1. Table 3. Clinical data and relative expression of miR-371a-3p in serum of healthy men.

## Data Availability

No datasets were generated or analysed during the current study.
