# Supplementary material for: Expression of the novel serum biomarker of testicular germ cell tumours miR-371a-3p in serum of pregnant women: a case–control study
Source: Eur J Med Res. 2025 Aug 2;30:697. doi: 10.1186/s40001-025-02906-8 (PMC12317637; doi:10.1186/s40001-025-02906-8)
Supplement: Supplementary file 1 — Supplementary material 1. Table 1. Clinical data and relative expression of miR-371a-p in serum of non-pregnant women. Table 2. Clinical data and relative expression values of miR-371a-3p in serum of patients with germ cell tumours clinical stage 1. Table 3. Clinical data and relative expression of miR-371a-3p in serum of healthy men. [file 40001_2025_2906_MOESM1_ESM.docx]

**Supplementary Table 1.** Clinical data and relative expression of miR-371a-p in serum of non-pregnant women

| **Case ID** | **Age [years]** | **RQ** |
| --- | --- | --- |
| 1 | 21 | 0.000 |
| 2 | 22 | 0.000 |
| 3 | 22 | 0.000 |
| 4 | 23 | 0.000 |
| 5 | 24 | 0.000 |
| 6 | 18 | 0.000 |
| 7 | 29 | 0.000 |
| 8 | 38 | 0.000 |
| 9 | 46 | 0.000 |
| 10 | 44 | 0.000 |
| 11 | 35 | 0.000 |
| 12 | 35 | 0.000 |

RQ: relative quantity

**Supplementary Table 2.** Clinical data and relative expression values of miR-371a-3p in serum of patients with germ cell tumours clinical stage 1

| **Case ID** | **Age [years]** | **Histology [S/NS]** | **RQ** |  |
| --- | --- | --- | --- | --- |
| 1 | 33 | S | 14.520 |  |
| 2 | 21 | NS | 125.366 |  |
| 3 | 36 | S | 46.527 |  |
| 4 | 37 | S | 374.806 |  |
| 5 | 37 | S | 153.560 |  |
| 6 | 36 | S | 715.400 |  |
| 7 | 43 | NS | 14.910 |  |
| 8 | 40 | NS | 80.004 |  |
| 9 | 35 | NS | 1,227.920 |  |
| 10 | 37 | S | 1,594.620 |  |
| 11 | 53 | S | 96.360 |  |
| 12 | 22 | S | 239.130 |  |
| NS: non-seminoma; RQ: relative quantity; S: seminoma | | | | |

**Supplementary Table 3.** Clinical data and relative expression of miR-371a-3p in serum of healthy men

| **Case ID** | **Age [years]** | **RQ** |
| --- | --- | --- |
| 1 | 49 | 0.000 |
| 2 | 36 | 0.000 |
| 3 | 24 | 0.744 |
| 4 | 34 | 1.452 |
| 5 | 35 | 0.788 |
| 6 | 23 | 0.000 |
| 7 | 39 | 0.641 |
| 8 | 18 | 0.000 |
| 9 | 36 | 0.012 |
| 10 | 29 | 0.289 |
| 11 | 25 | 0.000 |
| 12 | 24 | 0.000 |

RQ: relative quantity
